# *Yucca schidigera* Improves Performance and Lowers Oocyst Counts in *Eimeria* Challenged Broilers

**DOI:** 10.3390/ani12131668

**Published:** 2022-06-29

**Authors:** Krzysztof Kozłowski, Peggy Vervenne-Zetteler, Paweł Konieczka, Łukasz Szymański, Anouk van Vilsteren

**Affiliations:** 1 Department of Poultry Science and Apiculture, Faculty of Animal Bioengineering, University of Warmia and Mazury in Olsztyn, 10-718 Olsztyn, Poland; kristof@uwm.edu.pl (K.K.); lukasz.szymanski@uwm.edu.pl (Ł.S.); 2 Jadis Additiva, 3111 PN Schiedam, The Netherlands; peggy.vervenne@jadis-additiva.com (P.V.-Z.); anouk.vilsteren@jadis-additiva.com (A.v.V.)

**Keywords:** *Yucca schidigera*, coccidiosis, *Eimeria*, performance, broiler, saponins

## Abstract

**Simple Summary:**

Coccidiosis, caused by protozoa of the *Eimeria* species, is one of the most common causes of intestinal problems in poultry. In addition to economic losses and animal welfare being jeopardized, emerging resistance of the parasites against chemical and ionophore anticoccidial treatments is lurking. There is a demand for (natural) alternatives to the current treatments. In this trial, a *Yucca schidigera* product was tested for effectiveness in *Eimeria*-challenged chicken. *Yucca schidigera* is a plant which is native to the Mojave Desert, Chihuahuan Desert, and Sonoran Desert of southeastern California, Baja California, New Mexico, southern Nevada, and Arizona. Adding *Yucca schidigera* abolished the effect of the challenge, resulting in comparable or better performance results compared to the unchallenged birds. Therefore, *Yucca schidigera* can be considered as an effective alternative for anticoccidial treatment in broilers.

**Abstract:**

Coccidiosis is one of the main challenges for the worldwide poultry industry, and several anticoccidial treatments have been used to fight these protozoa. Resistance of *Eimeria* parasites against anticoccidials—and the public opinion about these treatments—demands for alternatives. In this trial, we tested *Yucca schidigera* (500 g/T) as a natural alternative in broilers challenged with *Eimeria*. This treatment was compared to three other treatments: An unchallenged control, a challenged control, and a challenged anticoccidial (625 g/T) treatment with focus on performance, oocyst per gram counts (OPG), and lesion scores. Both the anticoccidial and the *Yucca schidigera* group showed significant improvement in body weight (2.150 and 2.058 vs. 1.998 and 1.971 kg). growth (60.2 and 57.6 vs. 55.8 and 55.1 g/d). and feed-conversion ratio (1.456 and 1.510 vs. 1.527 and 1.575), compared to both control groups. No significant differences were found between the treatments on OPG collection days 18 and 25. On day 35, lowest OPG counts were found in the unchallenged group (93), followed by the *Yucca schidigera* group (114), the anticoccidial group (243), and the challenged group (650). Adding *Yucca schidigera* abolished the effect of the challenge resulting in comparable (FCR) or better (ADG) performance results compared to the unchallenged birds. *Yucca schidigera* consistently showed lower numbers for OPG counts compared to the anticoccidial and challenged group. Therefore, *Yucca schidigera* can be considered as an effective alternative for anticoccidial treatment in broilers.

## 1. Introduction

For many years the poultry industry has suffered from production losses due to the intestinal infection coccidiosis caused by protozoa of the *Eimeria* species. Coccidiosis outbreaks are mainly attributed to intensive rearing conditions and the *Eimeria* oocysts being highly infectious. The economic losses have increased from estimated losses of USD 800- million per year for world’s commercial poultry producers [1] in the 1990s, to spending about GBP 7.7–13.0 billion (at 2016 prices) in only seven countries on prophylaxis, treatment, and production losses due to avian coccidiosis [2]. The most significant problem is the less visible subclinical coccidiosis representing nearly three-quarters of the total economic costs. This is characterized by suboptimal flock performance due to increased feed intake and reduced body weight gain (BWG) [3].

In addition to economic losses, and animal welfare being jeopardized, emerging resistance against treatments is lurking [4]. Several chemicals and ionophore anticoccidial feed additives have been used widely since 1939 to fight these parasites [5]. However, although coccidiostats are often rotated, the poultry industry is challenged by resistance of the *Eimeria* parasites to these treatments. Moreover, public opinion demands alternatives for these treatments, and preparations based on natural ingredients are increasingly popular due to consumer preferences [6,7,8,9].

One of the proven alternatives is the 100% plant-based *Yucca schidigera* [10,11,12,13]. *Yucca schidigera* is an herbaceous plant of the lily family, native to the deserts of the southwestern United States and northern Mexico. This plant was used in traditional medicine by Native Americans to treat a variety of ailments, and yucca products are used in a number of applications. Yucca powder and yucca extract are used as animal feed additives, as discussed in detail by Cheeke and Otero (2005) [14]. *Yucca schidigera* contains two active compounds, which are the glycocomponent fraction, and the steroidal saponin fraction [15]. The glycocomponents can directly bind ammonia, while steroidal saponins have surface active properties, improve digestion, reduce emissions, and have an antiprotozoal effect. In relation to that, it has also been shown that *Yucca schidigera* in poultry improves feed conversion and production performance, lowers ammonia emissions and in- house and litter odors, and has antioxidant and immunomodulatory properties [15,16,17,18,19].

The antiprotozoal effect can help in poultry with the reduction in coccidiosis due to *Eimeria*. The active ingredients responsible for this effect are steroidal saponins, which forms a complex with the cholesterol present in protozoal cell membranes, causing damage to the integrity of the membrane resulting in cell lysis and the protozoa to die (Cheeke et al., 2006) [10]. Due to this effect of the saponins, the *Yucca schidigera* can be used as an alternative to coccidiostats. Previous research showed that the use of *Yucca schidigera* resulted in similar or better results on oocyst count and lesions in the gut compared to commonly used ionophores (narasin, salomycin, monensin, and nicarbazin) (Trejo Castro, 2002) [11]. Oviedo et al. (2003) [12] tested the effect of *Yucca schidigera* in a challenge model and compared it to (or in combination with) anticoccidials (monensin and salinomycin); an anti-infective (Stafac); Bacitracin Methylene Disalicylate (BMD), with or without the use of a vaccine on performance; OPG count; lesion scores; and anticoccidial index. The single product and combinations with *Yucca schidigera* showed the best results on growth, FCR (Feed conversion ratio) and oocyst index. No differences were found for feed intake and lesion scores. Alfaro et al. (2007) [13] tested *Yucca schidigera* against both coccidiostats (monensis d0–21, salinomycin d22–35) and a vaccine, and found a potential synergistic effect between the vaccine and *Yucca schidigera*. Oelschlager (2019) [20] tested two dosages of a Yucca-derived saponin product in a challenge model and found no differences in performance between the Yucca treatment and the infected birds, and better performance for the uninfected birds. Oocyst counts were not improved with the use of the product. Because results are not consistent, further research is required.

Therefore, the objective of this study was to evaluate the potential effects on production performance and coccidiosis of *Yucca schidigera* compared to two control treatments (unchallenged and challenged with *Eimeria*) and one of the most effective ionophore anticoccidials on the market (Maxiban) [21] in a 35-day broiler trial.

## 2. Materials and Methods

### 2.1. Ethical Statement

All procedures in the present study were evaluated and approved by the Local Animal Care and Ethics Committee in Olsztyn (UWM), Poland (Resolution No. 12/2022 from 16 March 2022), and were performed in accordance with the principles of the EU (recommendation 2007/526/CE) and the Polish Law on Animal Protection.

### 2.2. Experimental Design and Management

A total of 504 unsexed Ross 308 one day old chicks were divided over 4 treatments at random while ensuring the same starter weights between treatments. Every treatment consisted of 7 pens (7 replicates) with 18 birds each, resulting in 126 birds per treatment. The 4 treatments were: (1) unchallenged control; (2) challenged control; (3) challenged anticoccidial group receiving 625 g/T Maxiban (combination of narasin and nicarbazin, provided by Elanco, Greenfield, IN, USA); and (4) challenged *Yucca schidigera* (*Yucca s*.) group provided with 500 g/T of Yucca Plus^TM^, (high-quality *Yucca schidigera* product containing at least 10.5% steroidal saponins, provided by Jadis Additiva, Schiedam, The Netherlands) in the feed. The dosages used in this experiment are the recommended dosages of the supplier. The 3 groups that were challenged received a 40 times overdose of Avelon vaccine, containing *Eimeria* acervulina, *Eimeria* brunetti, *Eimeria* maxima, *Eimeria* necatrix, and *Eimeria* tenella strains, into the crop on day 14 and 21 of the trial.

The birds were kept in floor pens, 28 pens in total and 1.15 m^2^ each, with netting walls to avoid migration, and with pelleted straw as bedding material. The challenged and unchallenged treatments were separated by plastic screens. The trial was conducted in a house with windows. The house was provided with artificial programmable lights and climate, automated electric heating and forced ventilation. The heating and light program was in accordance with the Ross Broiler Management Manual [22]. The total length of the trial period was 35 days, which is a normal duration for challenge trials [11,23,24,25,26].

### 2.3. Applied Experimental Challenges

The broilers were challenged into crop with an Evalon vaccine (40 times recommended dose) on day 14 and 21 of the trial. The coccidia vaccine was administered directly into the crop with the use of a cannula. The vaccine was provided by AVIPOINT Poultry (Avipoint, Olsztyn, Poland) Surgeons, and contained *Eimeria* acervulina (003), *Eimeria* maxima (013), *Eimeria* mitis (006), *Eimeria* praecox (007), and *Eimeria* tenella (004) strains of *Eimeria*. The degree of intestinal mucosa damage was evaluated by a veterinarian (a poultry disease specialist) based on anatomopathological changes at 28th day of age according to the previously described protocol [27].

### 2.4. Diets and Feeding Scheme

A two-phase feeding scheme was applied (day 0–21 starter period and day 22–35 grower period) and the birds received feed and water ad libitum. A basal meal was made from which all treatment diets were produced and fed as meal. The nutritional value of all diets corresponded to the nutrient requirements of Ross 308 broiler chickens [22]. The composition and nutritional value of basal feed mixtures is shown in Table 1. The basal diets were analyzed for crude protein, crude fiber, crude fat, dry matter, and ash. Diets were produced by Agrocentrum Sp. z o.o. Feed mill (Pisz, Poland), who provided the feed to the trial site of University of Warmia and Mazury in Olsztyn (Poland)—where the experiment was conducted.

### 2.5. Measurements

Body weight of broilers (pen basis), and feed consumption was measured at day 1, 21, and 35. Mortality was recorded and animals that died or were removed from the trial were also weighed, feed-fed until selection date was included in the FCR, and reason for selection noted. Based on these results, the feed conversion ratios (FCR) over the experimental periods 1–21, 22–35, and 1–35 days were calculated. Average weight gain per bird for each period (AWG) = F–S, where F is the average weight of the live birds in the pen on the weighing day and S is the average weight of the live birds in the pen at the first weighing.

The average feed consumed per bird, per day, for the period was calculated according to following formula: $x=\frac{A}{\left(B*C\right)+\left(D\right)}$ where: 

A = Total feed consumed per pen for that period;

B = Number of surviving birds;

C = Day of study or the number of days for that period;

D = The sum of the days on which birds which have died (+ culled) were alive.

The average feed consumed for the period was calculated as follows: X × Y, where Y = the number of days in the period studied and the feed-conversion ratio for the period was calculated as total feed consumed for the period in each replicate divided by the total weight gain for this period (considering the WG of the dead and culled birds).

Lesion scores from 3 birds per pen were scored at day 28. OPG count scoring was performed at day 18, 25, and 35 for all pens. DM in litter was measured at the end of study by taking five samples of about 0.1 kg from 5 different points (4 corners and center of the pen, excluding the areas directly under the heater and the drinker) which were mixed, and moisture was determined after being in a forced air oven at 75 °C for 48 h.

### 2.6. Statistical Analysis

Body weight (pen means), BWG, feed intake, and FCR were analyzed separately by ANOVA as a randomized block experiment to compare dietary treatments. Livability was transformed into arc sin for statistical evaluation, but values (mean ± SD) are presented in %. Because of a big variability of OPG results, the outliers analysis was performed.

Analysis of variance and comparisons of mean differences between groups for all analyzed means were performed by ANOVA and Duncan test using Statistica for Windows Operating System (version 13.3), (Informer Technologies, Inc., Los Angeles, CA, USA). *p* (probability) was obtained from the F value in the ANOVA table. Significance was declared at *p* ≤ 0.05.

## 3. Results

### 3.1. Feed

Both the starter and grower basal meals were analyzed. These results are given in Table 2. The analyzed values are in line with the calculated values.

### 3.2. Growth Performance

Body weight and gain, feed intake, feed conversion ratio and livability are shown in Table 3. Both the anticoccidial and the *Yucca s*. group showed significant improvement in body weight, gain, and feed conversion ratio compared to the control groups (unchallenged and challenged). No significant differences were found for feed intake or livability. The unchallenged group had a significantly better feed conversion ratio compared to the challenged group.

### 3.3. OPG Count

The average number of oocysts per gram, feces per collection day, and dry matter (DM) in the litter is given in Table 4. Even though the unchallenged control was not vaccinated or inoculated still some shedding was seen. No significant differences were found between the treatments on collection days 18 and 25 due to large variation, although the *Yucca s.* showed numerically the lowest OPG counts on these days. On day 35 lowest counts were found in the unchallenged group, followed by the *Yucca s*. group and the anticoccidial group. Highest OPG counts were found for the challenged group, with significantly higher numbers compared to the three other treatments. In addition, the OPG counts of the *Yucca s*. group did not significantly differ from the unchallenged control on any of the collection days. 

### 3.4. Lesion Scores

The lesion scores taken from the intestine of three broilers per pen on day 28 are given in Table 5. No statistical analysis was performed on these data. Numerically, only the unchallenged birds differ from the other treatments. The anticoccidial group has numerically the highest number for total lesion scores (TLMS). Only the challenged and the *Yucca s.* group showed occurrence for *Eimeria* tenella, while the other groups showed only *Eimeria* acervullina and *Eimeria* maxima lesions.

## 4. Discussion

To perform a good trial for evaluation of the effect of different treatments against coccidiosis, it is important to include a challenge. In this trial it is clear that the challenge with the 40 times overdose of Avelon vaccine was successful, since the difference between OPG counts of the challenged, and unchallenged group was significant. The unchallenged group also had some shedding indicating that the separation of the challenged and unchallenged group was not completely successful.

Growth performance results show that *Yucca s.* is capable to improve BW, ADWG, and FCR compared to both the challenged and the unchallenged control groups. These findings are in line with earlier research of Oviedo et al. (2003) [12] and Alfaro et al. (2007) [13], which both found that *Yucca schidigera* (alone or together with a coccidiosis vaccine) improved growth and FCR in broilers. Sahoo et al. (2015) [16], Sun et al. (2017) [17], and Khaskheli et al. (2020) [28] also found that *Yucca schidigera* supplementation could improve growth performance in unchallenged broilers, probably since the steroidal saponins also improve digestion in general. However, earlier research of Oelschlager et al. (2019) [20] showed no significant difference in performance for the *Yucca schidigera* group with other treatments. This difference can probably be explained by the fact that *Yucca schidigera* sources can differ in their quality and composition, more specifically in the amount of steroidal saponins which are responsible for the antiprotozoal effect and effect on digestion. In the studies of Oviedo et al. (2003) [12] and Alfaro et al. (2007) [13], the same product was used as for this present trial, while the product used in the research of Oelschlager et al. (2019) [20], Micro-Aid, was not, and saponin content was not specified. In addition to this, the challenge model differs (strains of *Eimeria* vs. a 40-times vaccine overdose). For these reasons the results cannot be compared. However, in animal species in general it has been previously reported that *Yucca schidigera* is beneficial to animal performance and to reduce ammonia and fecal odors in animal excreta [11].

The livability of the birds in this trial was in general very good, with livability being only 1.4–1.6% numerically reduced compared to the unchallenged control group (which was set at 100%). If we compare this to previous research, it was also found that mortality is unrelated to treatment [20]. However, there are also other trials in broilers without coccidiosis challenges, which do show lower mortality due to *Yucca schidigera* [18,19].

The DM in litter was also evaluated in this study on day 35 and although there were no significant results, it is interesting to discuss the fact that *Yucca schidigera* was able to keep it at the same level as in the unchallenged group, while the challenged group had a lower DM, and therefore more moisture in the litter. This is interesting, since the combined effect of *Yucca schidigera* in reducing moisture in the litter and binding ammonia can help with reducing foot pad dermatitis. Sahoo et al. (2015) [16] also found to numerically lower moisture content in litter. Further research is necessary to evaluate this effect, since—to our knowledge—no research has been performed yet on food pad dermatitis for *Yucca schidigera*, and it shows great promise.

The OPG counts show that there are no significant differences on collection days 18 and 25, although *Yucca schidigera* did numerically reduce OPG counts in these days. On day 35 the OPG counts are significantly different between treatments, while the OPG counts between the *Yucca schidigera* group and unchallenged birds were not different. This shows that *Yucca schidigera* can keep OPG counts low even in coccidiosis-challenged birds. This has also been found in previous research in broilers [11]. The lesion scores did not show the same trend as the OPG counts, since only the unchallenged birds differed (numerically) from the other treatments. In the challenged birds—the Maxiban group and the *Yucca schidigera* group—lesions were found, but without real differences between them. Therefore, it can be stated that OPG counts and lesion scores do not seem to be correlated. This is in line with research of Shojaei (2014) [29] and Thakur et al., (2015) [30], where one strain of broilers had higher OPG counts but lower lesion scores [18] or OPG counts were significantly different while lesion scores were not [30]. However, the combination of performance, OPG counts, and lesion scores do give a complete picture of the differences between treatments for this present trial. 

The difference in performance between the anticoccidial treatment and the *Yucca schidigera* might be due to the difference in dosage of the products (625 g/T vs. 500 g/T). Further explanation could be that the combination of the chemical and ionophore anticoccidial in Maxiban has a higher effect compared to other single coccidiostats in the market. In previous research it was found that *Yucca schidigera* was able to improve or maintain growth performance, and reduce or maintain about the same OPG counts compared to single coccidiostats such as salinomycin, narasin, and monensin [11,12]. Furthermore, it can be expected that a 100% natural product such as *Yucca schidigera* has difficulties in outperforming a synthetic product such as Maxiban, especially when the dosage is lower. However, it would be interesting to test if the results would improve with a higher dose of *Yucca schidigera*, or when other anticoccidials are used as a comparison—especially since we know that the use of anticoccidials probably need to be reduced more and more in the future. Therefore, further research is necessary—for example, a trial with other treatments and including dose–response treatments for *Yucca schidigera*.

## 5. Conclusions

This research showed that animal performance was improved for both the *Yucca schidigera* and anticoccidial treatment. Adding *Yucca schidigera* abolished the effect of the challenge resulting in comparable (FCR) or better (ADG) performance results compared to the unchallenged birds, although the anticoccidial group still performed slightly better. *Yucca schidigera* showed consistently lower numbers for OPG counts compared to both the anticoccidial and challenged group. It may be concluded that *Yucca schidigera* can be considered as an effective alternative for anticoccidial treatment in broilers, while also having other benefits on performance and ammonia reduction. Hence, *Yucca schidigera* can be safely used in broiler-rearing for higher economical return, and reducing the costs associated with coccidiosis problems.

## Figures and Tables

**Table 1:** Calculated composition and nutritional value of the basal starter and grower diets, % (as-fed basis).

Specification	Starter (0–21 d)	Grower (22–35 d)
Feed composition		
Wheat	35.00	40.00
Soybean meal	30.17	26.75
Corn	26.55	20.62
Rapeseeds	3.00	5.00
Soya oil	1.90	4.49
NaCl	0.07	0.18
Limestone	1.17	1.13
Mono calcium phosphate	0.88	0.74
Methionine	0.32	0.24
L-Lysine	0.34	0.23
L-Threonine	0.07	0.09
Ronozyme P	0.01	0.01
Ronozyme WX	0.02	0.02
Vit-Min-Premix ^1^	0.50	0.50
Calculated nutrient density		
ME, kcal/kg	2960	3150
Crude protein	21.50	20.30
Lysine	1.30	1.15
Methionine	0.62	0.53
Methionine + Cystine	1.00	0.90
Threonine	0.83	0.80
Calcium	0.85	0.80
Phosphorus available	0.33	0.30
Sodium	0.05	0.09

^1^ Provides per kg feed: IU: vit. A. 10,000, vit. D3 4500; mg: vit. E 80, vit. B1 1.5, vit. B2 5, biotin 0.12, vit. B6 2.5, vit. B12 0.02, vit. K3 3, nicotinic acid 50, folic acid 1.1, pantothenic acid 14, choline 200, betaine 160, Mn 120, Zn 100, Se 0.35, Cu 20, Fe 40, J 3, Ca 0.6 g., phytase 1000 FTU.

**Table 2:** Proximal analysis of starter and grower basal diets, % (as-fed basis).

Basal Meal	Dry Matter	Crude Protein	Crude Fat	Crude Fiber	Crude Ash
Starter (0–21 d)	89.15	21.10	4.68	3.12	4.83
Grower (22–35 d)	89.91	20.04	6.27	3.38	4.22

**Table 3:** Growth performance of unchallenged and challenged chickens for the period between 1 to 35 day of life.

	BW, 1 Days kg	BW, 35 Days kg	ADWG G	ADFI G	FCR kg/kg	Livability %
Treatment						
Unchallenged	0.043 ± 0.001	1.998 ± 0.040 ^c^	55.8 ± 1.1 ^c^	91.1 ± 1.9	1.527 ± 0.028 ^b^	100.0 ± 0.0
Challenged ^1^	0.043 ± 0.001	1.971 ± 0.04 ^c^	55.1 ± 1.2 ^c^	92.8 ± 2.2	1.575 ± 0.022 ^c^	98.4 ± 4.2
Anticoccidia ^2^	0.043 ± 0.001	2.150 ± 0.054 ^a^	60.2 ± 1.5 ^a^	93.5 ± 2.2	1.456 ± 0.057 ^a^	98.4 ± 2.7
*Yucca s.* ^3^	0.043 ± 0.001	2.058 ± 0.055 ^b^	57.6 ± 1.6 ^b^	92.3 ± 3.2	1.510 ± 0.037 ^b^	98.6 ± 2.6
SEM	<0.001	0.015	0.439	0.463	0.010	0.001
*p*	0.937	<0.001	<0.001	0.336	<0.001	0.627

Notes: no. replicates = 28 (7 replicates of 18 birds/treatment); SEM = Standard Error Mean; BW = body weight; ADWG = mean daily weight gain; ADFI = mean daily feed intake; FCR = feed/gain. Values in same columns with no common superscript (^a,b,c^) are significantly different (*p* ≤ 0.05). ^1^ The broilers were challenged into crop with an Evalon vaccine (40 times recommended dose) on day 14 and 21 of the trial. The coccidia vaccine contained *Eimeria* acervulina (003), *Eimeria* maxima (013), *Eimeria* mitis (006), *Eimeria* praecox (007), and *Eimeria* tenella (004) strains of *Eimeria.* ^2^ Anticoccidial group receiving 625 g/T feed Maxiban (combination of narasin and nicarbazin). ^3^ With 500 g/T of high-quality *Yucca schidigera* product containing at least 10.5% steroidal saponins.

**Table 4:** Average number of oocysts per gram feces-OPG and dry matter in litter (%) of unchallenged and challenged chickens.

Treatment	OPG			DM in Litter
	18th Day	25th Day	35th Day	35th Day
**Unchallenged**	451 ± 270	300 ± 161	93 ± 45 ^a^	49.38 ± 7.78
**Challenged ^1^**	61,171 ± 100,146	2921 ± 2593	650 ± 196 ^c^	43.52 ± 5.48
**Anticoccidial ^2^**	19,744 ± 22,784	2043 ± 1291	243 ± 137 ^b^	48.92 ± 6.47
*Yucca s*. ^3^	3925 ± 3311	1433 ± 2735	144 ± 78 ^ab^	49.04 ± 7.71
SEM	9923.4	401.8	46.5	1.302
*p*	0.115	0.114	<0.001	0.349

Notes: no. replicates = 28 (7 replicates of 18 birds/treatment); SEM = Standard Error Mean. Values in same columns with no common superscript (^a,b,c^) are significantly different (*p* ≤ 0.05). ^1^ The broilers were challenged into crop with an Evalon vaccine (40 times recommended dose) on day 14 and 21 of the trial. The coccidia vaccine contained *Eimeria* acervulina (003), *Eimeria* maxima (013), *Eimeria* mitis (006), *Eimeria* praecox (007), and *Eimeria* tenella (004) strains of *Eimeria.* ^2^ Anticoccidial group receiving 625 g/T feed Maxiban (combination of narasin and nicarbazin). ^3^ With 500 g/T of high-quality *Yucca schidigera* product containing at least 10.5% steroidal saponins.

**Table 5:** Lesion scores of unchallenged and challenged chickens at 28 days of age.

*Eimeria* Strain	Unchallenged	Challenged ^1^	Anticoddidial ^2^	*Yucca s.* ^3^
*Eimeria* acervullina	0.130	0.210	0.928	0.571
*Eimeria* maxima	0.070	1.285	0.857	0.857
*Eimeria* tenella	0	0.070	0	0.286
TLMS	0.200	1.565	1.785	1.714

^1^ The broilers were challenged into crop with an Evalon vaccine (40 times recommended dose) on day 14 and 21 of the trial. The coccidia vaccine contained *Eimeria* acervulina (003), *Eimeria* maxima (013), *Eimeria* mitis (006), *Eimeria* praecox (007), and *Eimeria* tenella (004) strains of *Eimeria*. ^2^ Anticoccidial group receiving 625 g/T feed Maxiban (combination of narasin and nicarbazin). ^3^ With 500 g/T of high-quality *Yucca schidigera* product containing at least 10.5% steroidal saponins. Notes: no. replicates = 28 (7 replicates of 18 birds/treatment) for lesion scores 3 birds per each replicate were examined. TLMS = total mean lesion score.

## Data Availability

The data presented in this study are available upon reasonable request from the corresponding author.
